# Redescription of *Dynoides elegans* (Boone, 1923) (Crustacea, Isopoda, Sphaeromatidae) from the north-eastern Pacific

**DOI:** 10.3897/zookeys.646.10626

**Published:** 2017-01-17

**Authors:** Regina Wetzer, Gracie Mowery

**Affiliations:** 1 Research and Collections Branch, Natural History Museum of Los Angeles County, 900 Exposition Boulevard, Los Angeles, California 90007 USA; 2 University of Southern California, Los Angeles, CA 90089 USA

**Keywords:** Isopoda, Sphaeromatidae, Dynoides, California, East Pacific, intertidal

## Abstract

*Dynoides elegans* (Boone, 1923) from southern California is reviewed, redescribed, and figured. The original species description did not include figures, making it difficult to attribute individuals to the species. *Dynoides saldanai* Carvacho and Haasmann, 1984 and *Dynoides crenulatus* Carvacho & Haasman, 1984 from the Pacific Coast of Mexico and *Dynoides brevicornis* Kussakin & Malyutina, 1987, from Furugelm Island, Peter the Great Gulf in the Sea of Japan, appear morphologically more similar to each other than to western Pacific species. A large pleonal process is present in about half of the *Dynoides* species, but is absent in this north-eastern Pacific clade and the north-western Pacific *Dynoides brevicornis* and *Dynoides brevispina*. *Dynoides dentisinus* Shen, 1929 possess a large pleonal spine. It is known from China, Japan, and Korea and is introduced in San Francisco Bay; it can be easily distinguished from *Dynoides elegans* by the presence of a pleonal process in the former. A key to the Pacific West Coast *Dynoides* is provided.

## Introduction

The genus *Dynoides* Barnard, 1914 was erected for *Dynoides serratisinus* Barnard, 1914 from Natal and Mozambique (Kensley 1978) and currently has 20 accepted species (WoRMS, World Register of Marine Species, Bruce and Schotte 2013). A complete synonymy for the genus was provided by Li (2000). In the north-eastern Pacific three species are known: *Dynoides crenulatus* Carvacho & Haasman, 1984, *Dynoides saldanai* Carvacho & Haasmann, 1984, both from the Pacific Coast off the Oaxacan State of Mexico, and the species redescribed here, *Dynoides elegans* (Boone, 1923). Additionally, *Dynoides dentisinus* Shen, 1929 originally described from the coast of North China also occurs in San Francisco Bay. Kussakin and Malyutina (1987) described *Dynoides brevicornis* from Furugelm Island, Peter the Great Gulf in the Sea of Japan, (north-eastern Pacific). Additional species occur in the western Pacific (Japan, Korea, China, Singapore, India, and Australia).

Pearl Lee Boone described several new isopod genera and species from the California coast in her 1923 paper, all without figures. She erected the new genus *Clianella* Boone, 1923 for *Clianella elegans* from La Jolla, California based on six specimens collected in 1915 “from bunches of mussels along the outer ledge of rocks north of Scripps Institution of Biological Research.” Of these she designated a male holotype and two male paratypes which are part of the United States National Museum of Natural History collections (Cat. Nos. 50421 and 1422085) and which were examined here. The other three paratypes were donated to the Scripps Institution of Biological Research (SIO). The SIO specimens could not be found in the SIO collections in 2016 and are presumed lost (pers. comm. Collection Manager, Harim Cha). Additionally, Boone included a single adult male specimen collected from “Point White, San Pedro, California, May 18, 1919, by Mr. E.P. Chace and donated to the U. S. National Museum” (USNM Cat. No. 1422085).

The individual designated by Boone as the holotype has previously had pleopods 1–4 removed. Some pleopods were recovered floating in the vial containing the specimen. One paratype had several broken pereopods, and its dorsum is cracked. The second paratype was complete and in as good a condition as can be expected of a 97-year-old specimen. Permission was granted for dissection and this individual is figured here.

The composition of *Dynoides* and its relationship to *Clianella* was reviewed by Li (2000) and the generic name *Clianella* placed into junior synonymy. The genus *Dynoides* is distinguished by “presence of pleotelsonic slit that may or may not have an anterior lobe and internal teeth; a penial process basally fused for half its length and an appendix masculina elongate, twice as long as the endopod and strongly reflexed” (Li 2000). Species of the genus are known from intertidal habitats.

## Abbreviations

LACM – Natural History Museum of Los Angeles County; LMU – Loyola Marymount University; MBPC – Marine Biodiversity Center; USC – University of Southern California; USNM – United States National Museum, Smithsonian Institution; RS – robust seta/e; PMS – plumose marginal setae; SEM – scanning electron microscopy. Latitudes and longitudes denoted with “~” are approximate and estimated from Google Earth.

## Material and methods

Descriptions are based on the male paratype and additional material as noted. Specimens examined have USNM or LACM catalog numbers. Numbers preceded by “RW” are field station numbers. Collections so labelled are readily retrieved in the LACM collections. Setal terminology broadly follows Watling (1989). We provide images of additional material from White Point (Boone’s “Point White”) and Pt. Fermin. Both localities are on the Palos Verdes Peninsula less than 5 km apart. Additionally, we examined material from Santa Catalina, San Cruz Islands (California Channel Islands) and Cedros Island (Baja California Norte, Mexico).

Specimens are prepared for SEM as described in Wall et al. (2015). Drawings were made with the aid of a *camera lucida* and illustrations were electronically “inked” with Affinity Designer, Serif Labs. Whole body illustrations were made with a Wild M5D stereo dissecting scope. Appendages were illustrated by dissecting off the appendage and placing them in glycerol on a depression slide and then imaged using a Nikon Labophot-2 compound scope. Specimens were measured with a micrometer. The lengths given in the “Material Examined” are of the largest specimen of each species and sex. Not all specimens were measured. If a length is provided and multiple specimens were present in a lot, the length refers to the largest specimen. In all species mature males appear larger than females, but body lengths for mature adults are similar. Males have broader and longer uropods than females, which contributes to this illusion.

Molecular data were generated for this species according to the protocols described in Wetzer et al. (2013). Voucher specimens are held in the LACM collections.

## Taxonomy

### Key to the north-eastern Pacific species of *Dynoides* of the North American West Coast

This key is based on adult ♂ characters. Also note that weak pereon tubercles are visible only with SEM and not necessarily evident with light microscopy.

**Table:** 

1	Pleon with elongate dorsal process, produced into prominent, triangular, posteriorly pointed, conical process, extending to anterior margin of the teardrop-shaped pleotelsonic slit; pleotelsonic slit with strong crenulate margins	***Dynoides dentisinus***
–	Pleon without triangular, posteriorly pointed, conical process; pleotelsonic slit teardrop- or heart-shaped; pleotelsonic slit without or with only weak crenulate margins	**2**
2	Pleon without broad shelf-like ridge. Pleotelson in form of bilobed dome. Pleotelsonic slit with parallel sides; base of slit teardrop-shaped	***Dynoides crenulatus***
–	Pleon with broad shelf-like ridge. Pleotelson vaulted, without bilobed dome. Pleotelsonic slit elongated teardrop- to completely heart-shaped. Base of pleotelsonic slit with prominent tubercle barely overlapping base of slit	**3**
3	Pleotelsonic slit heart-shaped, without crenulate slit margins. Antennule with 9 flagellar articles, only 7 distalmost articles with aesthetascs. Antenna article 5 length 1.4 × width. Penes distal apex distinctly acute; basal half fused; apex pitch fork-shaped. Pleopod 3 exopod with suture	***Dynoides saldanai***
–	Pleotelsonic slit elongated teardrop- to heart-shaped, slit with weak crenulate margins. Antennule with 14 or more flagellar articles, only 12 distalmost articles with aethetascs. Antenna article 5 length 2.2 × width. Penes distal apex rounded and blunt; basal third fused; apex tuning fork-shaped. Pleopod 3 exopod without suture	***Dynoides elegans***

### Dynoides

Taxon classificationAnimaliaIsopodaSphaeromatidae

Barnard, 1914

#### Type species.

*Dynoides serratisinus* Barnard, 1914: 408; from South Africa by monotypy.

#### Remarks.

A diagnosis and comprehensive synonymy was provided by Li (2000). Readily recognizable characteristics include cephalon longer than broad, penes fused along proximal half of the length. Appendix masculina elongate, twice as long as endopod, strongly reflexed. Uropodal rami broad, lamellar, and subequal in length. The genus presently has ~20 species, is intertidal to shallow water, and is most speciose in the northern Pacific with twelve species. Additional species occur off Brazil, South Africa, Sri Lanka, and Australia (Li 2000). At present, the relationships between species remain unassessed.

### Dynoides elegans

Taxon classificationAnimaliaIsopodaSphaeromatidae

(Boone, 1923)

Figures 1 , 2 , 3 , 4 , 5 , 6 , 7 , 8

Clianella elegans Boone, 1923: 153; Kussakin and Malyutina 1993: 1174.
Dynoides elegans . Li 2000: 138.

#### Material examined.

HOLOTYPE ♂ (7.04 mm), California, San Diego County, La Jolla, Scripps Institution for Biological Research, ~32.27°N ~117.61°W, 23 Oct 1915, USNM 50421 [RW16.020] designated by Boone.

2 ♂ PARATYPES (6.03, 5.36 mm, smaller specimen dissected and figured), same data as holotype, USNM 1422085.

Non-type material: 1 ♂ (6.16 mm), California, Los Angeles County, White Point, San Pedro, ~33.715°N ~118.314°W, 18 May 1919. Coll. E.P. Chace, USNM 50422 [RW16.022].

2 ♂ (largest ♂ 5.36 mm, 2^nd^ male used for SEM), plus 8 non-gravid females, subadults and juveniles, California, Los Angeles County, Palos Verdes Peninsula, Pt. Fermin, shore at Paseo del Mar, ~0.5 mi. W of Gaffey Street, 33.71°N 118.30°W, mid-low intertidal, chipping overhanging rock with hammer and *Phragmatopoma* tubes on underside of rock, 0.99 m. Fixed and preserved in 95% ethanol. 27 Mar 2004. Sta. #2. Coll. R. Wetzer, N.D. Pentcheff, and LMU students. RW04.030. LACM-MBPC 17829.

#### Additional material examined.

1 male (5.36 mm), 3 ?females/subadults, 13 juv., Pt. Fermin, shore at Paseo del Mar, ~0.5 mi. W of Gaffey Street, eastern end of beach, ~33.71°N ~118.3°W, mid intertidal, scraping live barnacles off deeply crenulated rock shelf, fixed and preserved in 95% ethanol. 13 Jun 2006. Coll. R. Wetzer. RW06.063. LACM-MBPC 17831.

1 male (5.35 mm), 5 subadults, 2 juv., Los Angeles County, Santa Catalina Island, Big Fisherman Cove, in front of USC Wrigley Institute, 33.44°N 118.48°W, algal scrapings, ca. 1-2 ft. below low water, fixed and preserved in 95% ethanol. 7 Apr 2006. Acc. No. F.P.2.2006-6. Coll. N.D. Pentcheff, N.L. Bruce, R.Wetzer. RW06.006. LACM-MBPC 17830.

2 subadult males (largest individual 5.4 mm), 2 juvenile specimens, and posterior half of a gravid female, Santa Catalina Island, Avalon Harbor, ~33.35°N ~118.33°W, either rock or artificial substrate, subtidal scrapings, 3.05 m. Probably fixed in formalin, stored in 95% ethanol. 1 May 2011. Sta. 406. Coll. LACSD, rcvd. from D. Cadien. RW12.212. LACM-MPBC 17832.

9 specimens (largest 7.37 mm), Santa Barbara County, Santa Cruz Island, Pelican Bay, ~34.035°N ~119.703°W, under *Mytilus* beds, 18 Jul 1939. Coll. W.G. Hewatt. RW16.019. USNM 86407, Acc. No. 154967.

1 male (6.83 mm), Pacific, Mexico, Baja California Norte, Cedros Island, South Bay, Sta. 288-34, 10 May 1934. RW16.028. USNM 252317, Acc. No. 128938.

2 specimens (3.9 mm and 4.0 mm) photographed live, Los Angeles County, San Pedro, White Point, 33.72°N 118.32°W, rocky intertidal, hand, preserved in 95% ethanol. 23 Jun 2016. Coll. A. Wall, J. Wall, K. Omura, N.D. Pentcheff, L. Harris. RW16.051. LACM-MBPC 16919.

#### Description of male.

**Figure 1.**
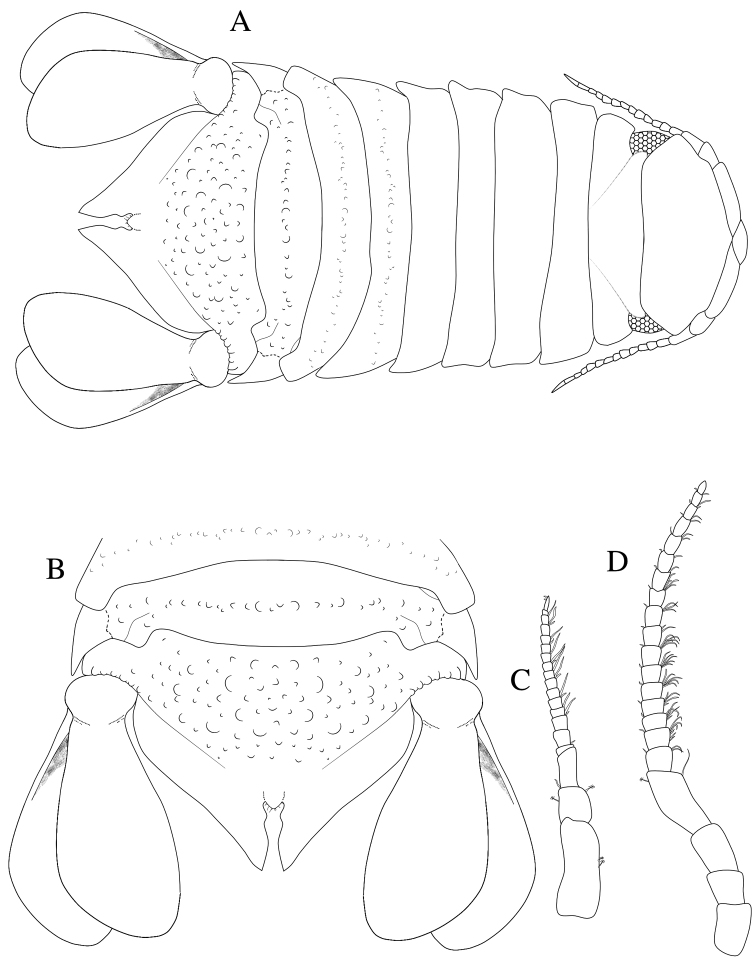
*Dynoides elegans*. ♂ Holotype. USNM 50421. **A** dorsal **B** pleotelson. ♂ Paratype. USNM 1422085. All from left **C** antennula **D** antenna.

*Body* length 2.4 × width; pereonites 1–5 smooth, pereonite 1 medially very slightly raised, pereonites 6–7 with very small tubercles; pleotelson covered with small tubercles; pleotelson length 1.2 × width, anterior of pleotelsonic sinus with prominent rounded tubercle barely overhanging base of sinus, sinus walls straight-sided, finely crenulate, and slightly raised. Coxal margins with setae appearing membranous, *membrana cingula*, (Figures 1A, B; 5B, C; 7A, B).

*Antennula* peduncle article 1 length 3.6 × width, anterior medial margin with 2 palm setae; article 2 as long as wide, inferior distal margin with 1 palm seta, superior margin with 1 palm seta; article 3 length 2.2 × width, proximal margin with 1 simple seta; flagellum with 14 articles, 12 distalmost articles with aesthetascs (Figure 1C). *Antenna* reaching anterior margin of pereonite 3; article 5 length 2.2 × width, flagellum with 17 articles (Figure 1D). *Clypeus* and *labrum* as in Figure 5E, F.

**Figure 2.**
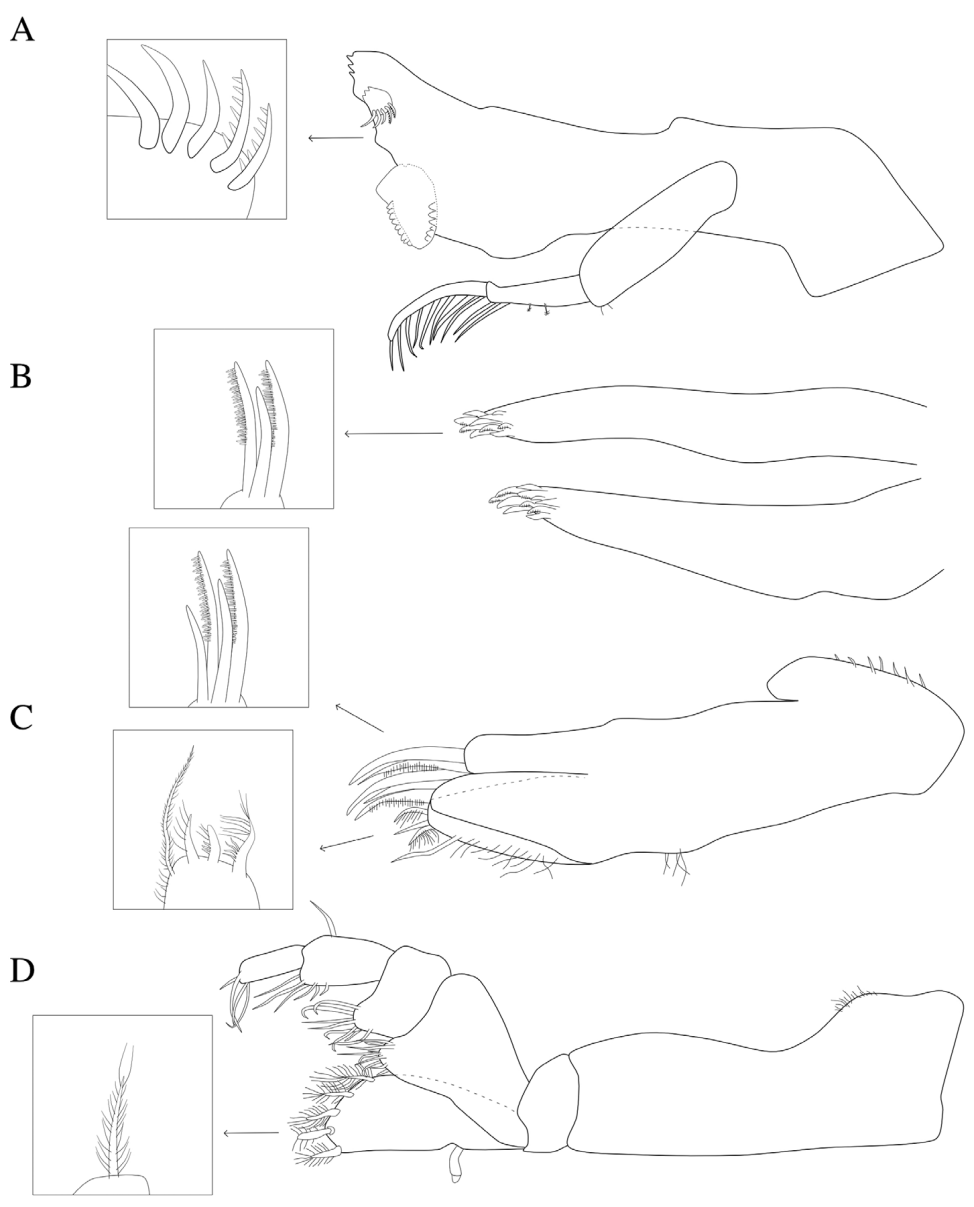
*Dynoides elegans*. ♂ Paratype. USNM 1422085. All mouthparts from left. **A** mandible **B** maxillula **C** maxilla **D** maxilliped.

*Left mandible* incisor with 3 cusps; lacinia mobilis with 3 cusps; lacinia mobilis spine row comprised of 2 serrate and 3 simple spines; crushing surfaces strongly ridged; mandibular palp article 1 with 2 minute setae; article 2 with 2 palm setae and 2 plumose setae; article 3 with long, plumose setae (Figure 2A). *Maxillula* mesial lobe with about 7 spines; lateral lobe with about 10 spines (Figure 2B). *Maxilla* mesial lobe with 2 simple setae and 3 plumose RS on gnathal surface; middle lobe with 2 simple setae and 2 pectinate RS; lateral lobe with 2 pectinate RS (Figure 2C). *Maxilliped* endite distal surface with 5 plumose setae; distomesial margin with 1 coupling hook; palp article 2 distal apex with 9 long, (some broken) simple RS; article 3 distal apex with 8 long, simple RS; article 4 distal apex with 6 long, simple RS, lateral distal angle with 1 long, simple RS; article 5 distal apex with 4 long, simple RS (Figures 2D; 5F).

**Figure 3.**
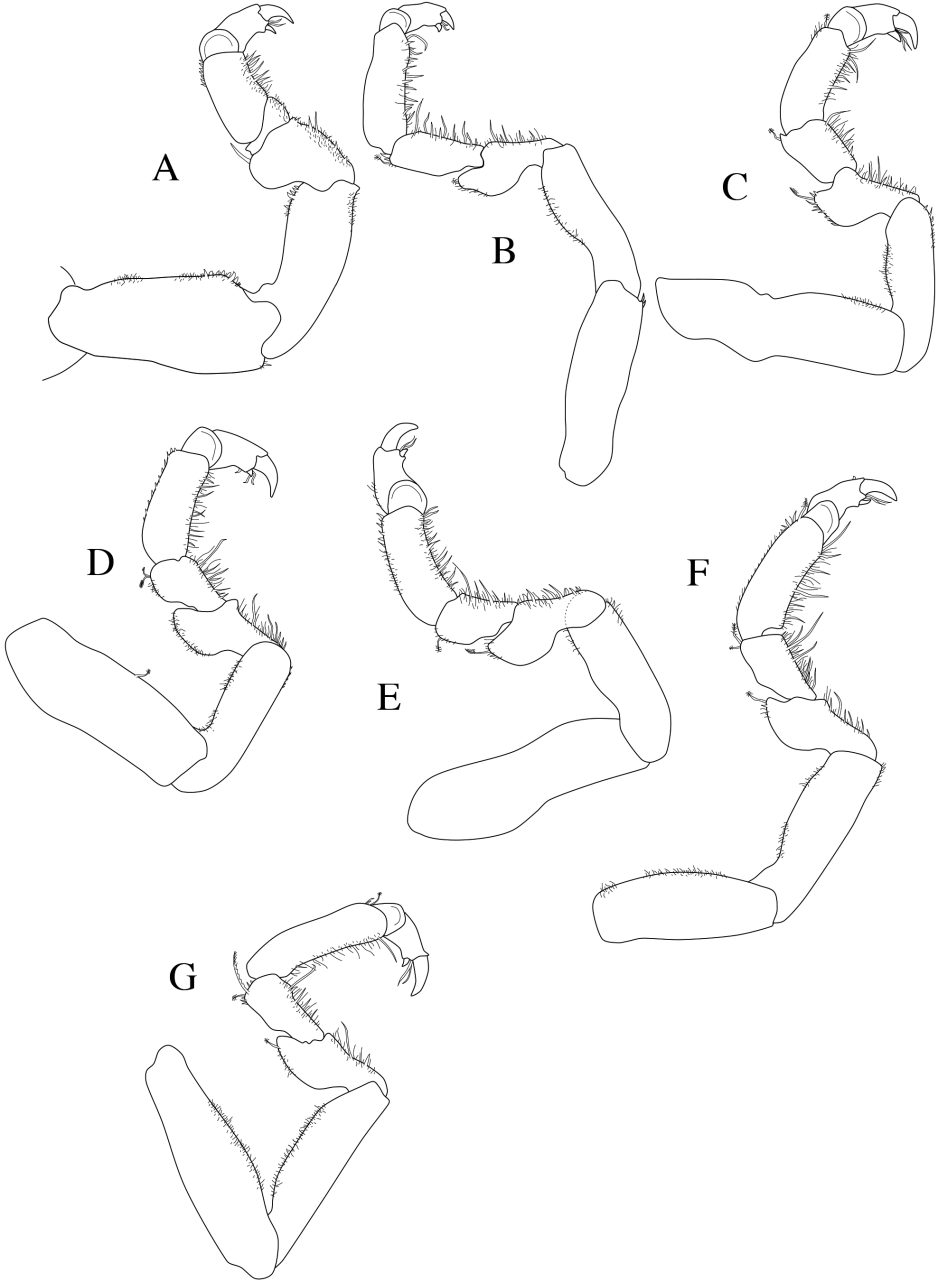
*Dynoides elegans*. ♂ Paratype. USNM 1422085. All appendages from left. **A** pereopod 1 **B** pereopod 2 **C** pereopod 3 **D** pereopod 4 **E** pereopod 5 **F** pereopod 6 **G** pereopod 7.

*Pereopods 1–7* (Figures 3A–G; 5F) all with one strong secondary unguis on the dactyl, ambulatory, and similar; merus, carpus, and propodus inferior margins more setose than superior margins (as figured). *Pereopod 1 basis* length 2.3 × width; *ischium* length 2.8 × width. *Pereopod 2 basis* length 3.6 × width; *ischium* length 3.1 × width. *Pereopod 1* more stout than *pereopods 2–7*. *Pereopod 7 basis* length 3.4 × width, *ischium* length 4.0 × width.

*Penial process* length 2.3 × basal width, basal third fused (Figure 5G).

**Figure 4.**
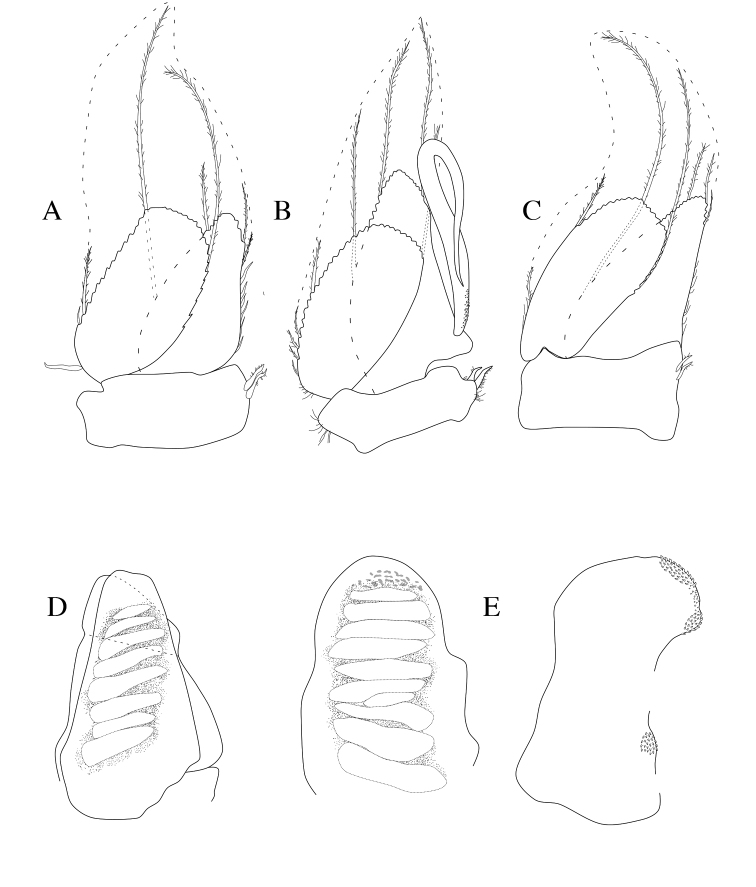
*Dynoides elegans*. ♂ Paratype. USNM 1422085. All appendages from left. **A** pleopod 1 **B** pleopod 2 **C** pleopod 3 **D** pleopod 4 **E** pleopod 5 (endopod and exopod separated).

**Figure 5.**
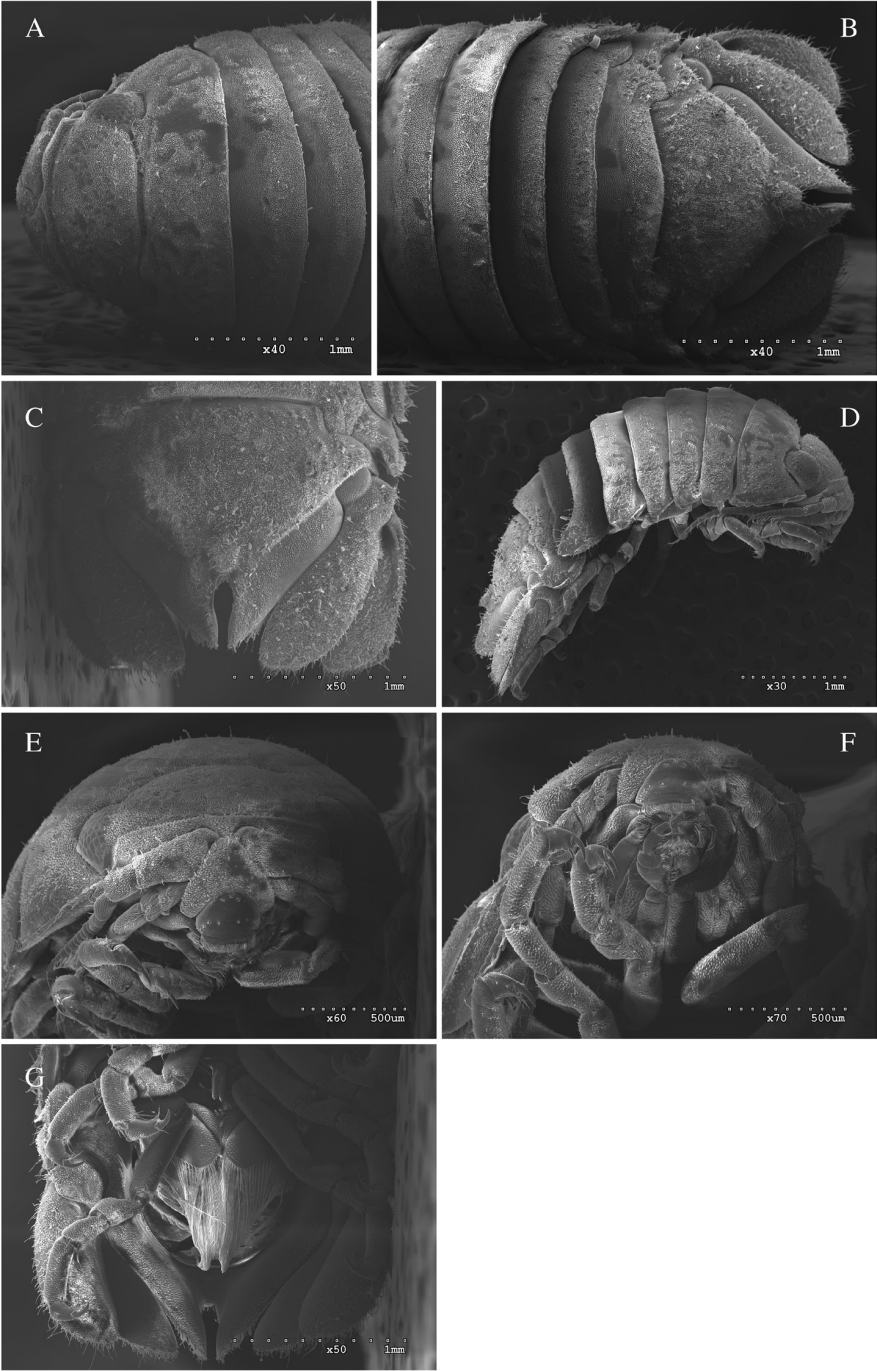
*Dynoides elegans*. ♂, non-type. LACM-MBPC 17829. **A** anterior dorsal **B** posterior dorsal **C** pleotelson **D** lateral **E** clypeus, labrum **F** mouthfield **G** ventral with penes.

*Pleopod 1* peduncle length 2.3 × width with 2 coupling hooks (Figure 4A); PMS extending to posterior margin of pleonal cavity (Figure 5G). *Pleopod 2* peduncle length 3.2 × width with 2 coupling hooks, *appendix masculina* proximally slightly swollen, distally narrowing, basal mesial margin with scales, distal third doubled back on proximal half (Figure 4B). *Pleopod 3* peduncle length 2.0 × width with 2 coupling hooks (Figure 4C). *Pleopods 1–3* exopods and endopods with PMS as figured (note: not all drawn, but indicated). *Pleopod 4* endopod and exopod subequal, exopod with transverse suture (Figure 4D). *Pleopod 5* endopod and exopod subequal, endopod length 1.4 × width with one distal scale patch and one smaller submedial scale patch, exopod length 1.6 × width (Figure 4E).

*Uropod* exopod proximolateral margin rolled, weakening distally; in the adult ♂ holotype (USNM 50421) and 2 adult ♂ paratypes (USNM 1422085) uropods extend well beyond posterior margin of pleotelson (as figured in Figure 1B), but do so otherwise only in the largest males (see Figures 5B; 7A, B).

#### Description of female.

**Figure 6.**
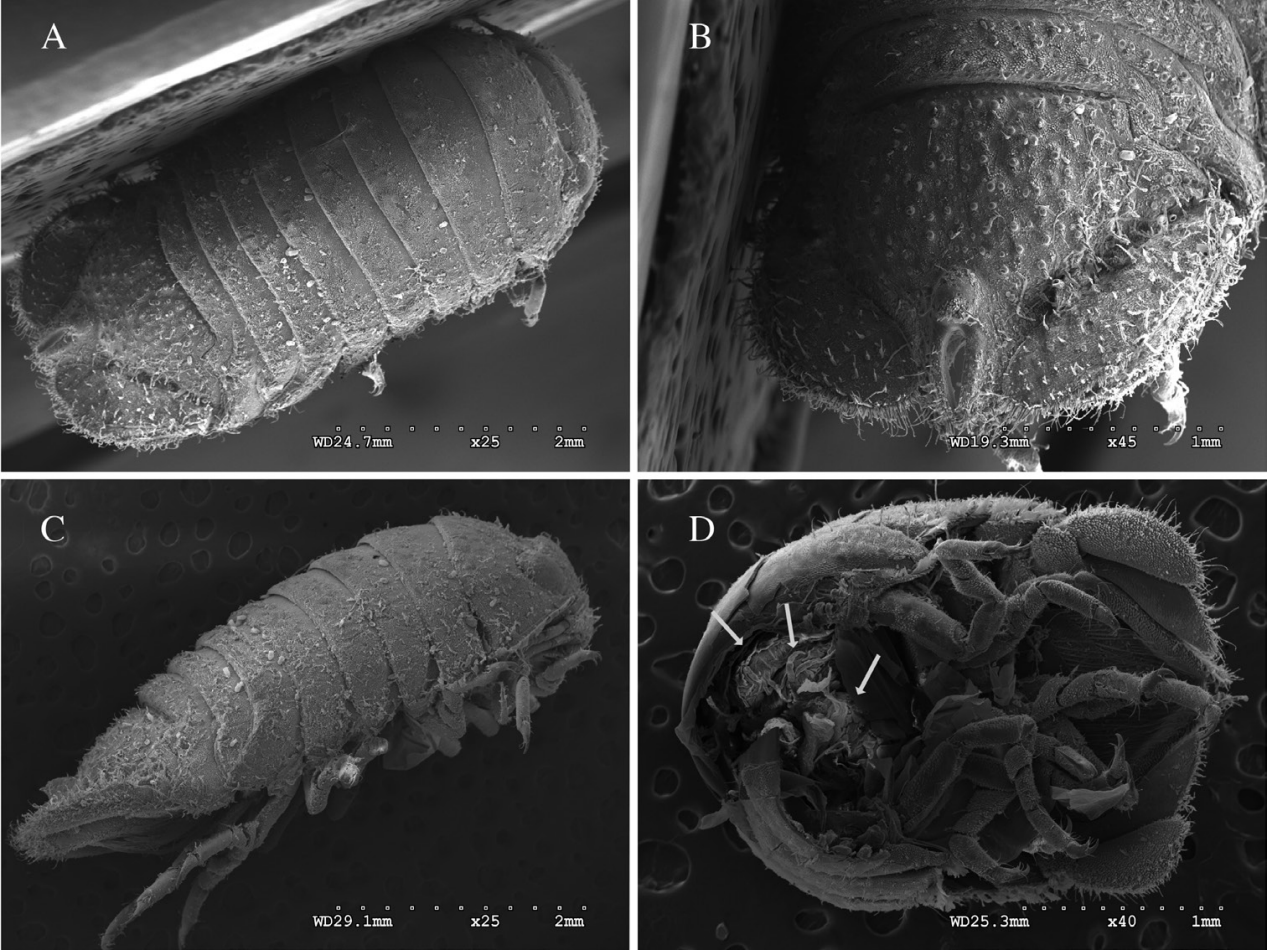
*Dynoides elegans*. ♀, non-type. USNM 86407. **A** dorsal **B** pleotelson **C** lateral. ♀, non-type. LACM-MBPC 17832 **D** posterior half of gravid individual with mancas and ventral view of pleotelson.

*Body* length 2.2 × width; (Figures 6A, B, C). Dorsal ornamentation as in the male. *Pleotelson* length 1.2 × width. *Uropodal* endopod longer than exopod, endopod just barely extending to posterior margin of pleotelson (Figure 6D). Dorsally uropodal exopod proximolateral margin weekly rolled, tapering to an evenly rounded distal margin. Gravid female estimated with 8–12 mancas. Figure 6D is the posterior half of female broken open exposing 3 mancas.

**Size.** Largest ♂ to 7.37 mm, largest ♀ to 5.4 mm.

**Figure 7.**
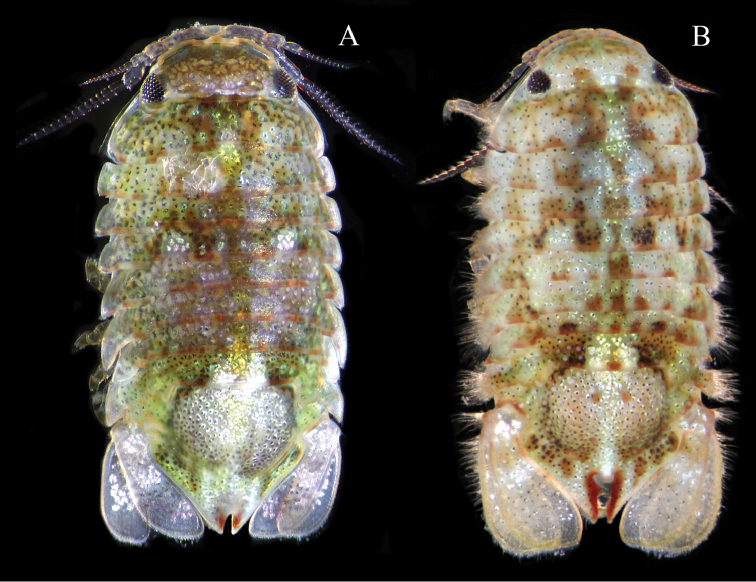
*Dynoides elegans*. ♂, non-type. LACM-MBPC 16919. Photographs of live specimens by Leslie Harris. **A** (3.9 mm) and **B** (4.0 mm).

**Color.** When alive brightly colored, individuals highly varied (Figure 7A, B). When preserved in ethanol, specimens quickly become pale buff to whitish. Bright red coloration outlining pleotelsonic slit fades last.

#### Distribution.

California: San Diego to Santa Barbara Counties.

#### Molecular data.

Both 18S-rDNA and 16S-rDNA were generated from the same individual from Pt. Fermin (RW04.030), GenBank numbers JF699541, and KU248214, respectively. Locality information is provided above in Material Examined. This specimen came from the same lot from which the SEM specimen in Figure 4 was prepared.

#### Remarks.

**Figure 8.**
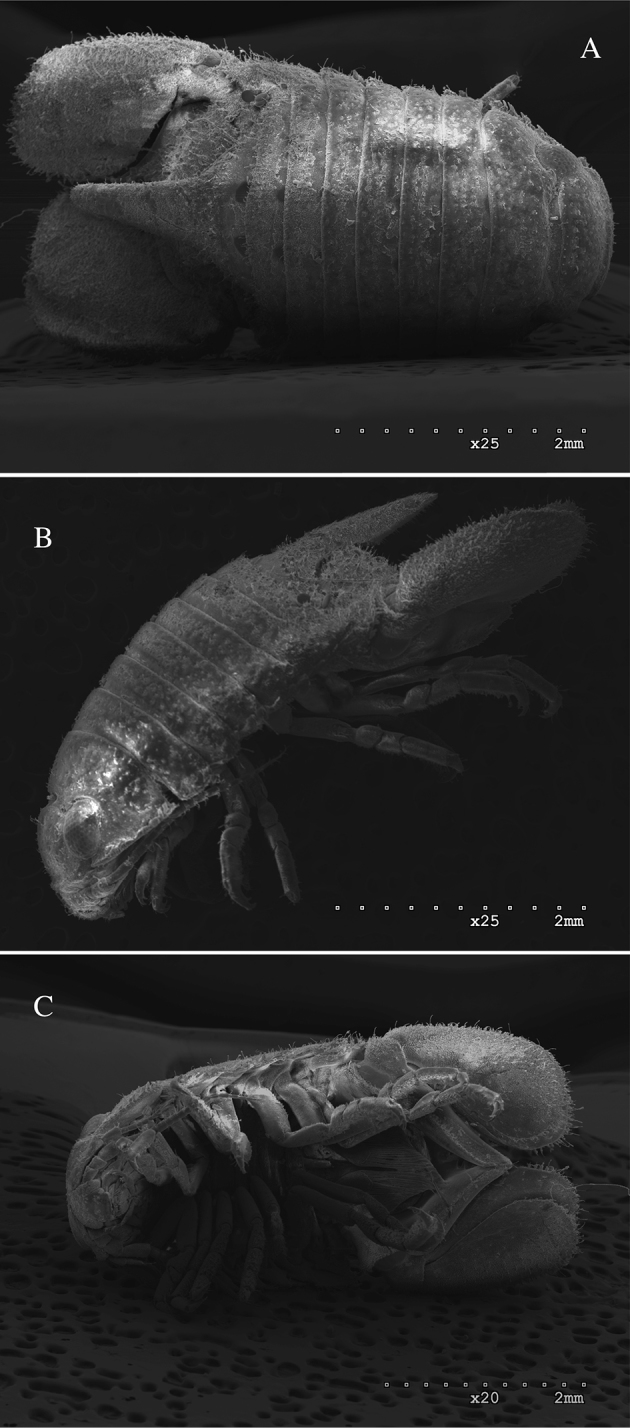
*Dynoides dentisinus*. ♂, **A** dorsal **B** lateral **C** ventral. California, Alameda County, San Francisco Bay, off Doolittle Road near Oakland Airport, 37.73°N 122.21°W, from low intertidal under rocks, associated with sponge, salinity 30 ppt, fixed and preserved in 95% ethanol. 5 Jun 2002. Coll. R. Wetzer, T. Haney, and S. Boyce. RW02.027. MBPC 17838.

*Dynoides elegans* is morphologically most similar to *Dynoides saldanai* and *Dynoides crenulatus* (Pacific, Mexico, Oaxaca). These three species are easily distinguished from *Dynoides dentisinus*. Adult male specimens of *Dynoides dentisinus* are more robust than those of *Dynoides elegans* and have a distinctive, prominent large process extending over the pleotelson (Figure 8A, B). The presence of a prominent pleonal process is polymorphic in *Dynoides* (Li 2000). The presence of a slit, sinus, notch, or foramen is also variable in the genus (Li 2000). A pleotelson slit of various shapes is present in all three eastern Pacific *Dynoides* and the north-western Pacific *Dynoides brevicornis* (Kussakin and Malyutina 1987).

A generic description of the “penes fused along proximal half of length” (Li 2000) is an easily recognizable character. In *Dynoides elegans* penes may be considered fused closer to proximal third of length (Figure 5G). Of all of the material available for examination, we had only a single broken gravid female (Figure 6D). Gravid females are clearly rare. This may be attributed to our poor sampling during brooding episodes, which remain unknown. No specimens collected during the months January/February, August/September, or November/December were available for examination.

*Dynoides elegans* is most similar to the Oaxacan species, *Dynoides saldanai*. They share pleonal characters which are known to change as individuals, especially males, mature. Penial processes, pleonal process, appendix masculina, pleotelson morphology and also pleotelson sinus morphology are characters that all change with age in males. A fully adult male (penes and appendix masculina developed) may not be at the final fully developed male stage, potentially with some further changes to the pleotelson morphology. We do know that in males the sinus will transition progressively from a simple slit to a quite complex structure. The body length of the *Dynoides elegans* type specimens range from 5.36 to 7.04 mm. The subtle changes in morphology are readily observed in Figure 7, represented by specimens from the same collecting event. Note uropodal development and progression of a simple pleotelsonic slit to a heart-shaped slit. The largest male examined was 7.37 mm (Santa Cruz Island), and its pleotelsonic slit approaches heart-shape. The largest *Dynoides saldanai* specimen is 4.45 mm in length and female 3.0 mm. The two male specimens in Figure 7A and 7B are 3.9 and 4.0 mm in length.

The figured male paratype (Figure 1A) has a body length 2.35 × width. We note that in all other specimens measured, adult male body length is closer to 2.2 × width. Non-empirical observations of this species and other Sphaeromatidae from the north-eastern Pacific indicate that sexually mature adult males reached larger body sizes in the past than they do today (RW pers. obser.). Specimens of *Dynoides elegans* collected before the 1940s are among the largest individuals in the examined collections, with the largest males exceeding 7 mm body length. The largest specimens come from the oldest collections (specimens collected between 1915–1939). It appears that fully developed males in the past attained larger body sizes than more recently collected individuals (e.g., 2004–2016). To quantify this, populations of individuals appropriate to determine statistical significance need to be evaluated.

*Dynoides elegans* is known from San Diego County to Santa Barbara County, with a single male specimen (USNM 252317) recorded from Cedros Island off the Pacific Baja California coast. The Cedros Island specimen is most similar to White Point and Pt. Fermin specimens (Los Angeles County). These localities are roughly 700 km apart. To definitively confirm that Cedros Island is the southernmost locality in the species range, additional specimens are needed. Appropriate material for molecular analysis would greatly contribute to our understanding of the morphological diversity within species (e.g., varying amounts of membrane-like setae on the coxal margins, refer to Figure 7), across populations, and allow us to determine whether *Dynoides saldanai* might be a junior synonym of *Dynoides elegans*. The *Dynoides saldanai* type series consists of 27 specimens: 2 adult males, 10 juvenile males, 11 females, and 4 undetermined junveniles. The male holotype (4.45 mm) and allotype were deposited at the Institute of Biology of the National Autonomous University of Mexico. The paratypes are alleged to be in the National Museum of Natural History in Paris (Carvacho and Hassmann 1984). Type numbers were not provided. When substantially more fresh material has been collected, it would be useful to clarify the status of *Dynoides saldanai* by comparisons with the type material and any other specimens attributed to *Dynoides saldanai* and *Dynoides elegans*. Not examining *Dynoides saldanai* types at this time does not effect the status of *Dynoides elegans*.

## Supplementary Material

XML Treatment for Dynoides

XML Treatment for Dynoides elegans
